# Characterization of Cwc2, U6 snRNA, and Prp8 interactions destabilized by Prp16 ATPase at the transition between the first and second steps of splicing

**DOI:** 10.1261/rna.079886.123

**Published:** 2024-09

**Authors:** Jadwiga Meissner, Katarzyna Eysmont, Katarzyna Matylla-Kulińska, Maria M. Konarska

**Affiliations:** 1 IMol, Polish Academy of Sciences, 02-247 Warsaw, Poland; 2 ReMedy-International Research Agenda Unit, 02-247 Warsaw, Poland

**Keywords:** spliceosomal catalytic center, transition-state conformation, Prp16, U6 snRNA, Prp8

## Abstract

The spliceosome performs two consecutive transesterification reactions using one catalytic center, thus requiring its rearrangement between the two catalytic steps of splicing. The Prp16 ATPase facilitates exit from the first-step conformation of the catalytic center by destabilizing some interactions important for catalysis. To better understand rearrangements within the *Saccharomyces cerevisiae* catalytic center, we characterize factors that modulate the function of Prp16: Cwc2, N-terminal domain of Prp8, and U6-_41_AACAAU_46_ region. Alleles of these factors were identified through genetic screens for mutants that correct *cs* defects of *prp16-302* alleles. Several of the identified U6, *cwc2*, and *prp8* alleles are located in close proximity of each other in cryo-EM structures of the spliceosomal catalytic conformations. Cwc2 and U6 interact with the intron sequences in the first step, but they do not seem to contribute to the stability of the second-step catalytic center. On the other hand, the N-terminal segment of Prp8 not only affects intron positioning for the first step, but it also makes important contacts in the proximity of the active site for both the first and second steps of splicing. By identifying interactions important for the stability of catalytic conformations, our genetic analyses indirectly inform us about features of the transition-state conformation of the spliceosome.

## INTRODUCTION

The spliceosome, a highly dynamic molecular machine composed of five small nuclear RNAs (U1, U2, U4, U5, and U6 snRNAs) and over 100 proteins (Burge et al. 1999; Wahl et al. 2009), is responsible for splicing of all pre-mRNAs. The spliceosome assembles on each intron de novo and requires extensive compositional and conformational remodeling of its small nuclear ribonucleoprotein (snRNP) subunits to create the functional catalytic center. Substrate recognition is facilitated by pairing interactions with snRNAs: The branch site (BS) is bound by U2 snRNA (Parker et al. 1987), whereas the 5′ splice site (5′SS) is initially recognized by U1 snRNA (Zhuang and Weiner 1986; Séraphin et al. 1988; Siliciano and Guthrie 1988), and subsequently, within the catalytic spliceosome, it interacts with U6-ACA within the conserved _47_ACAGA_51_ motif (Kandels-Lewis and Séraphin 1993; Lesser and Guthrie 1993b). The cryo-EM structures capture the spliceosome at various stages of splicing and depict its catalytic center as a stable, unchanged structure (Galej et al. 2016; Yan et al. 2016, 2017; Bertram et al. 2017a, b; Fica et al. 2017; Plaschka et al. 2017; Bai et al. 2018; Wan et al. 2019). In contrast, genetic data indicate that the catalytic center must change between the two catalytic steps of splicing to allow for the repositioning of substrates for catalysis (Madhani and Guthrie 1994; Mefford and Staley 2009; Eysmont et al. 2019).

Consecutive rearrangements of the spliceosome are facilitated by dedicated ATPases/RNA helicases of the DExH/D-box family, e.g., Prp5, Prp28, Brr2, Prp2, Prp16, Prp22, and Prp43, acting at different stages of the splicing pathway, from assembly through catalytic reactions, to the release of products and spliceosome recycling (Staley and Guthrie 1998). Thus, monitoring defects caused by ATPase alleles and identifying suppressors of these defects allow us to study conformational changes of the spliceosome at the selected transition in the splicing process. For example, Prp2 remodels protein interactions around the BS, facilitating the entry of the spliceosome into the first catalytic conformation (Kim and Lin 1996; Warkocki et al. 2009; Lardelli et al. 2010). Thus, *prp2* alleles inhibit entry into the first-step catalytic conformation. Prp16 facilitates the transition between the first and second catalytic steps (Schwer and Guthrie 1991, 1992; Burgess and Guthrie 1993; Query and Konarska 2004; Villa and Guthrie 2005). In *Saccharomyces cerevisiae*, a defective *prp16-302* allele carrying two point mutations, *R456K* + *G691R* in the ATPase domain, can serve as a convenient stage-specific marker that stabilizes the first-step conformation of the spliceosome, resulting in improved first-step catalysis but reduced progression to the second step. Thus, the cold-sensitivity (*cs*) defects of *prp16-302* reflect an inefficient exit from the first-step conformation. Suppressors of *prp16-302* are expected to destabilize the first-step conformation and facilitate the transition from the first-to-second steps of splicing.

**FIGURE 1.**
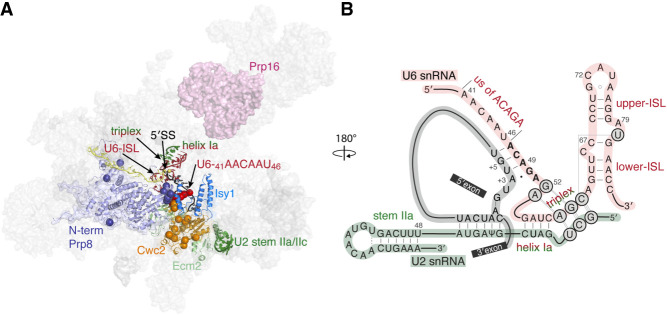
Structural representation of spliceosomal components involved in the first-to-second steps transition. (*A*) Structure of *S. cerevisiae* spliceosome immediately after branching (5LJ5) (Galej et al. 2016). Spliceosome factors involved in the transition between the first and second steps are shown in color: Prp16 (pink), Isy1 (blue), Ecm2 (light green), U6–U2 helix Ia and the catalytic triplex (red/green), U2 stem IIa/IIc (green), U6-ISL and U6-_41_AACAAU_46_ (red), Cwc2 (orange), and Prp8 N-terminal domain (purple). Location of *cwc2*, U6, and *prp8* alleles suppressing *prp16-302* growth defects described in this paper are marked by spheres. (*B*) Schematic representation of U6 (red), U2 (green), and pre-mRNA (gray) at the catalytic center of the spliceosome. Base-pair interactions altered by Prp16 action are depicted.

The RNA core of the catalytic center of the spliceosome involves sequences of U2, U6, and U5 snRNAs. Elements of U6 and U2 snRNAs form the catalytic triplex (Fica et al. 2014), which, together with the adjacent U6 intramolecular stem–loop (U6-ISL), coordinate metal ions for catalysis (Steitz and Steitz 1993) and through adjacent U2-_34_GUAGUA_39_ and U6-_47_ACAGA_51_ interactions, help to juxtapose the BS and 5′SS for catalysis. Despite the mostly unchanged structure of the RNA catalytic core seen in cryo-EM structures, multiple elements of U6 and U2 have been genetically identified to suppress the *cs* defects of *prp16* alleles, indicating that they destabilize structures of the first-step catalytic conformation of the spliceosome (U6-_41_AACAAU_46_: [Madhani and Guthrie 1994]; U2 stem IIa/IIc: [Hilliker et al. 2007; Perriman and Ares 2007]; U2/U6 helix Ia: [Mefford and Staley 2009]; catalytic triplex: [Fica et al. 2014]; U6-lower ISL: [Fig. 1B; Eysmont et al. 2019]). Although snRNA mutants are thought to destabilize the catalytic conformation in all these cases, the detailed mechanisms responsible for the improved transition to the second-step conformation are unknown.

Analysis of U6 alleles in the catalytic triplex and the adjacent ISL revealed the presence of two competing conformations that modulate the transition between the two catalytic steps (Eysmont et al. 2019). A cluster of U6 alleles in the region of _41_AACAAU_46_ (Fig. 3B) has been previously identified to suppress the *cs* phenotype of *prp16* alleles (Madhani and Guthrie 1994), thus improving transition to the second-step conformation. Because this cluster of alleles is adjacent to the U6-_47_ACAGA_51_ motif known to bind the 5′SS, we sought to understand its function better.

In addition to U2 and U6 alleles, multiple changes in protein components of the spliceosome have been identified to suppress defects of *prp16* alleles and are thus predicted to affect the conformational change between the catalytic steps (Fig. 1A). Deletion of *isy1*, a component of the NineTeen Complex (NTC), suppresses *prp16-302* defects and has been shown to genetically interact with mutations in the U6 upstream of the ACAGA region (Villa and Guthrie 2005). Deletion of NTC-associated protein *ecm2* suppresses *prp16-R686I* defects (van der Feltz et al. 2021). Additionally, biochemical analyses indicate that weakened binding of the first-step factor Yju2 bypasses the requirement of Prp16 for the transition to the second step (Chiang and Cheng 2013). Cwc2 protein, an NTC component (Ohi and Gould 2002), joins the spliceosome during the activation for the first step of catalysis, stabilizes intron:U6 interactions, and contacts the U6-_41_AACAAU_46_ region (McGrail et al. 2009; Rasche et al. 2012; Schmitzova et al. 2012; Hogg et al. 2014).

Prp8, a large, highly conserved spliceosomal protein, directly contacts all of the components of the catalytic center. Prp8 joins the spliceosome as a part of the U5·U4/U6 snRNP and remains in the spliceosome throughout the catalysis (for review, see Grainger and Beggs 2005). Previously, alleles within the Prp8 linker domain (1376–1649 aa), endonuclease domain (1653–1824 aa), and RNaseH-like domain (1839–2092 aa) that suppress the first- or second-step defects in splicing catalysis were described (Collins and Guthrie 1999; Liu et al. 2007). These alleles are thought to stabilize the first or the second catalytic conformations, although specific structural changes leading to this stabilization are not known. However, no *prp8* alleles capable of suppressing *prp16* alleles defects have been identified. The Prp8 N-terminal domain (1–884 aa) is the least well-studied part of this protein.

Manipulation of Prp16 function by using wt versus mutant alleles provides a sensitive tool for monitoring interactions at the catalytic center. Using the yeast *S. cerevisiae* system, we explore contributions of three different classes of suppressors of *prp16-302* growth defects to altering stability of the catalytic center. Alleles in Cwc2, U6, and Prp8 correct *prp16-302* defects by destabilizing the first-step catalytic conformation. All these alleles seem to act by destabilizing the 5′SS positioning for the first-step catalysis, whereas *prp8* alleles are likely to additionally alter catalytic interactions at the active site affecting both catalytic conformations.

## RESULTS

### A genetic screen to identify *cwc2* alleles that destabilize the first-step conformation

**FIGURE 2.**
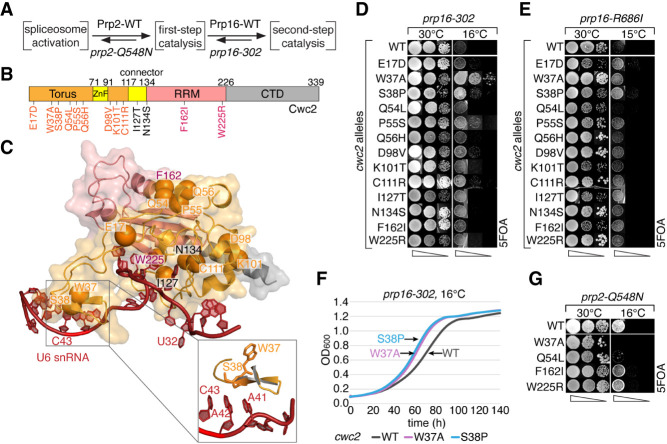
*cwc2* alleles identified in a genetic screen alter the first-step conformation, supporting transition to the second step. (*A*) Schematic of the catalytic phase of splicing. Conformations of the catalytic center are shown in brackets, and factors affecting transitions between these conformations are listed *above* (promoting) or *below* the arrows (inhibiting the transition). (*B*) Schematic of the yeast Cwc2 protein domain structure. Positions of alleles identified in the genetic screen are marked. (*C*) cryo-EM structure of Cwc2 (orange) and part of U6 snRNA (red) within the spliceosome complex C (5LJ5) (Galej et al. 2016). Positions of *cwc2* alleles are marked as spheres. The *inset* shows two Cwc2 amino acids, W37 and S38, adjacent to U6 C43-G39. In *prp16*Δ strain (yMK36), the *cs* growth phenotype of (*D*) *prp16-302* and (*E*) *prp16-R686I* alleles is suppressed by the identified *cwc2* alleles, as shown by spotting on 5-fluoroorotic acid (5FOA) plates. (*F*) OD_600_ measurements of yeast strains harboring *prp16-302* allele and a second copy of *cwc2*-*W37A* and *S38P* alleles show an improved growth as compared to Cwc2-WT at 16°C. (*G*) In *prp2*Δ strain (yAAH1915), *cwc2* alleles *W37A* and *Q54L* exacerbate the *prp2-Q548N cs* phenotype, as shown by spotting on 5FOA plates.

A pool of *CWC2* sequences generated by error-prone PCR mutagenesis was introduced into a *prp16*Δ yeast strain carrying the *prp16-302* allele (thus, in the presence of a wt genomic copy of *CWC2*) by homologous recombination (gap repair). To increase the yield of analyzed mutants, selection was carried out in parallel for two *CWC2* regions, spanning the segments corresponding to 1–159 aa and 159–339 aa. The screen identified 13 *cwc2* alleles that rescue the *cs* phenotype of *prp16-302* allele at 16°C (Fig. 2D), including a previously described *cwc2-W37A* allele in the Torus domain (Hogg et al. 2014). For the strongest *cwc2* alleles (*W37A* and *S38P*), suppression of *prp16-302* defects was confirmed by measuring growth rates in liquid media (Fig. 2F). Nearly all of the identified alleles (*cwc2-E17D*, *W37A*, *S38P*, *P55S*, *Q56H*, *D98V*, *K101T*, *C111R*, *F162I*, and *W225R*) also suppress *cs* defects of *prp16-R686I* allele at 15°C, implying the generality of these effects (Fig. 2E).

The majority of identified *cwc2* alleles are located in the Torus domain (*cwc2-E17D*, *W37A*, *S38P*, *Q54L*, *P55S*, *Q56H*, *D98V*, *K101T*, and *C111R*), two alleles are in the connector (*I127T* and *N134S*) and three are found in the RRM domain (*Y138F, F162I,* and *W225R*). The only Cwc2 region which did not yield any *prp16* suppressors is its C-terminal domain (CTD) known to interact with the WD40 domain of Prp19 (Fig. 2B; Ohi and Gould 2002). Spliceosomal cryo-EM structures position *cwc2-W37* and *S38* in proximity of the U6-_41_AAC_43_ and the N-terminal domain of Prp8 (Figs. 2C and 4A) (5LJ5, Galej et al. 2016).

Since *cwc2* mutants correct *prp16* defects by destabilizing first-step interactions, they also could inhibit entry into the first-step conformation, exacerbating defects of *prp2* alleles (Fig. 2A). To investigate whether *cwc2* alleles impact splicing at the spliceosome activation step, we looked for their genetic interactions with *prp2-Q548N*, a *cs* allele defective in the spliceosome activation (Fig. 2G; Wlodaver and Staley 2014). Both *cwc2-W37A* and *Q54L* exacerbate growth defects of *prp2-Q548N* at 16°C in the presence of a chromosomal copy of Cwc2, supporting the notion that they destabilize the first-step catalytic conformation.

Together, these data indicate that changes in Cwc2–U6 interactions destabilize interactions in the first-step catalytic center, inhibiting entry into and facilitating exit from the first-step spliceosomal conformation.

### Multiple mutations in the U6-_41_AACAAU_46_ motif correct defects of *prp16-302* allele, altering the transition between the first and second steps of splicing

Both biochemical and structural analyses indicate close contacts of Cwc2 with U6, including U6-ISL and the region upstream of ACAGA motif (McGrail et al. 2009; Rasche et al. 2012; Schmitzova et al. 2012; Hogg et al. 2014).

**FIGURE 3.**
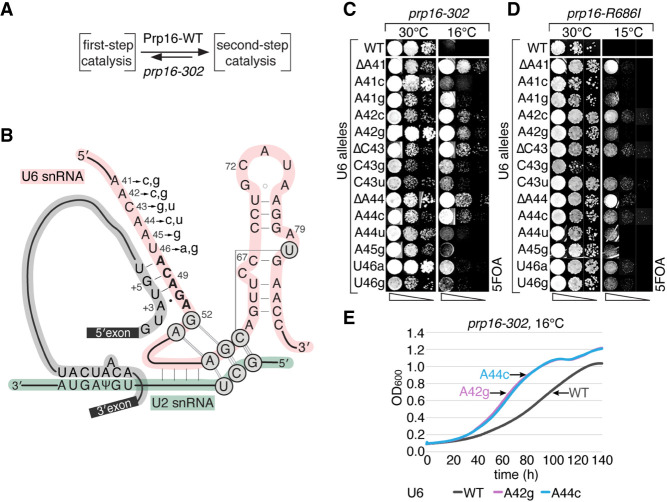
U6 alleles upstream of ACAGA motif support transition from the first-to-second steps of splicing. (*A*) Prp16 promotes transition from the first to the second step, whereas *prp16-302* allele inhibits this transition. (*B*) Schematic representation of U6 (red), U2 (green), and pre-mRNA (gray) at the catalytic center of the spliceosome. Mutations of U6 in the _41_AACAAU_46_ region are marked. In *prp16*Δ U6Δ strain (yCQ166), *prp16-302* (*C*) and *prp16-R686I* (*D*) alleles inhibit transition to the second step, exhibiting *cs* phenotypes suppressed by mutations within the U6 region _41_AACAAU_46_, as shown by spotting on 5FOA plates. (*E*) Growth curves (OD_600_) of yeast strains harboring *prp16-302* allele in combination with U6-A42g and A44c alleles exhibit improved growth, compared to U6-WT at 16°C.

Therefore, to test if Cwc2–U6 interactions functionally affect the first-step spliceosomal conformation, we analyzed the known U6 alleles located upstream of the ACAGA motif (Fig. 3B) that suppress *cs* defects of *prp16* alleles (Madhani and Guthrie 1994). To better understand the function of this U6 region, we prepared several single-point U6 alleles: ΔA41, A41c/g, A42c/g, ΔC43, C43g/u, ΔA44, A44c/u, A45g, and U46a/g, transformed them into a double-delete Δ*U6* Δ*prp16* yeast strain, and confirmed their ability to suppress *prp16-302 cs* defects at 16°C (Fig. 3C). Sensitive growth curve analyses confirm these suppression effects at 16°C; *prp16-302* strains carrying U6-A42g or A44c alleles grow faster than those carrying wt U6 (Fig. 3E). Similar suppression by U6 alleles was also observed for *prp16-R686I* allele at 15°C (except for U6-A41c and C43g alleles) (Fig. 3D). These findings confirm and extend earlier reports (Lesser and Guthrie 1993b; Madhani and Guthrie 1994), demonstrating that multiple mutations in the U6 region upstream of the ACAGA motif suppress growth defects of *prp16* alleles by destabilizing interactions essential for the first-step catalytic conformation.

### The N-terminal domain *prp8* alleles suppress *prp16-302 cs* growth defects

**FIGURE 4.**
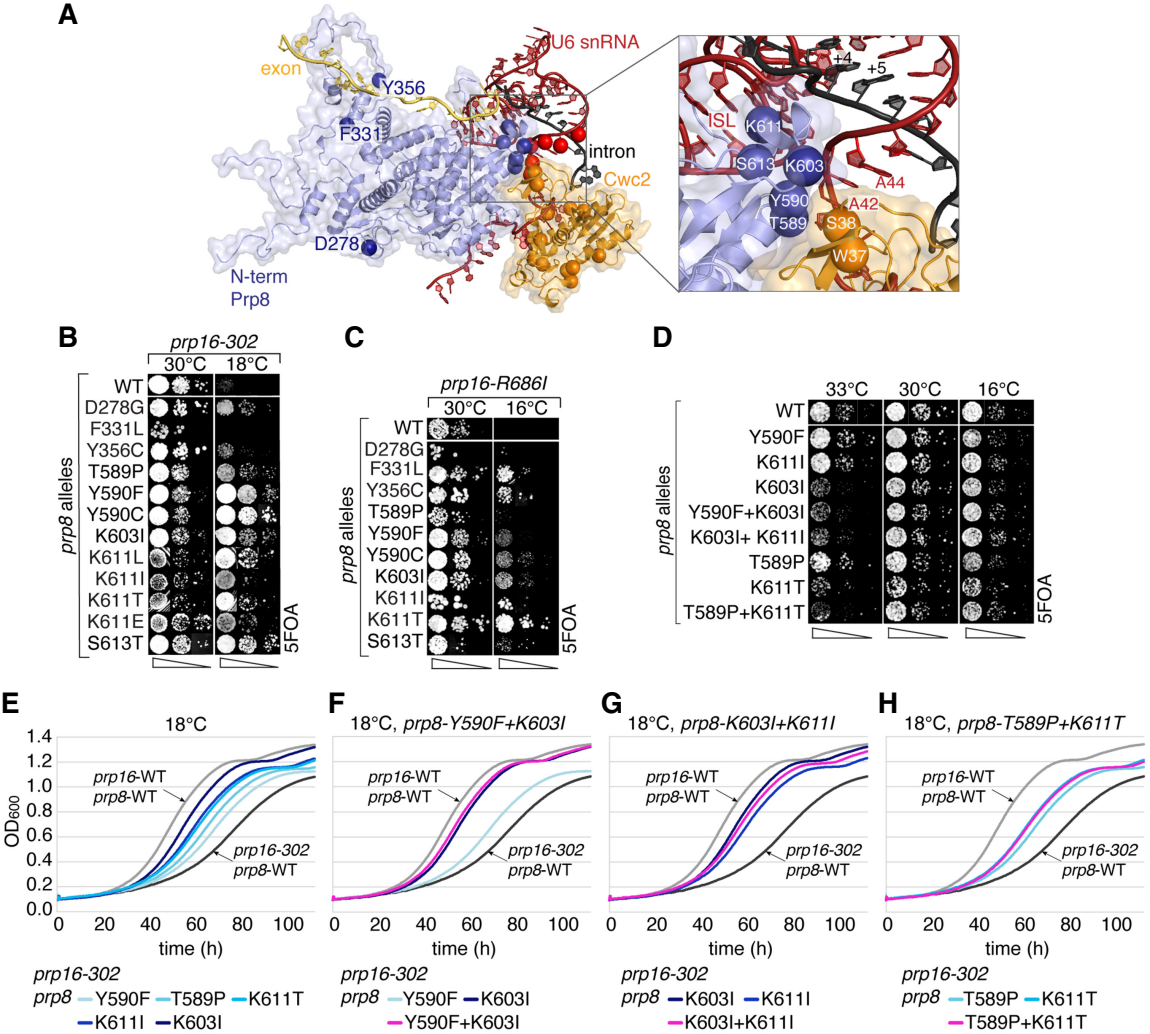
*prp8* alleles located in the N-terminal domain suppress *prp16* defects. (*A*) Prp8 N-terminal domain (purple), U6 (red), Cwc2 (orange), and pre-mRNA (intron-black, 5′ exon-yellow) as seen in complex C (5LJ5) (Galej et al. 2016). Locations of *prp8*, *cwc2*, and U6 alleles are marked by spheres. The *inset*: a region of close interactions between Prp8, Cwc2, and U6. In *prp16*Δ *prp8*Δ strain (yCQ06), the *cs* phenotype of *prp16-302* (*B*) and *prp16-R686I* (*C*) alleles is suppressed by mutations in the Prp8 N-terminal domain identified in the genetic screen. (*D*) Growth of combinations of *prp8* alleles *Y590F* + *K603I*, *K603I* + *K611I*, and *T589P* + *K611T* is not affected compared to single mutations. Suppression effects in *B*–*D* were monitored by spotting of the analyzed strains on 5FOA plates. (*E*) Growth curves (OD_600_) of yeast strains harboring *prp16-302* allele in combination with *prp8-Y590F*, *T589P*, *K611T/I*, and *K603I* alleles show improved growth compared to Prp8-WT at 18°C. Growth curves of combinations of *prp8* alleles *Y590F* + *K603I* (*F*), *K603I* + *K611I* (*G*), and *T589P* + *K611T* (*H*) demonstrate no additive growth improvement of strains carrying *prp16-302* allele at 18°C compared to single alleles.

To better understand interactions stabilizing the catalytic center, we next focused on Prp8, the largest component of the spliceosome that surrounds the RNA catalytic core. We carried out a genetic screen for *prp8* alleles within the Prp8 N-terminal domain that suppress *cs* defects of *prp16-302*. A library of PCR-mutagenized *prp8* segments corresponding to aa 4–838 was introduced by homologous recombination into *PRP8* plasmids in a *prp8*Δ, *prp16*Δ yeast strain harboring *prp16-302* allele. Resulting alleles were selected to improve strain growth at 18°C (Fig. 4B). This screen yielded multiple *prp8* alleles located close to the catalytic center: *prp8-T589P*, *Y590F/C*, *K603I*, *K611L/T/I/E*, *S613T*, as well as a few more distally positioned alleles: *D278G*, *F331L*, and *Y356C* (Fig. 4A) (5LJ5, Galej et al. 2016). These *prp8* alleles significantly suppress growth defects of *prp16-302* allele (Fig. 4B), and most of them (*prp8-F331L*, *Y356C*, *Y590C/F*, *K603I*, *K611I/T*, and *S613T*) also correct growth defects of *prp16-R686I*, demonstrating the generality of these effects (Fig. 4C). Suppression of *prp16-302 cs* defects by selected *prp8* alleles was also confirmed by growth curve measurements (Fig. 4E), with the strongest suppression phenotype observed for *prp8-K603I*. These results suggest that destabilization of N-terminal Prp8 domain contacts with the catalytic core facilitates exit from the first-step conformation. Based on their location, *prp8-T589P*, *Y590F/C*, *K603I*, *K611L/T/I/E*, and *S613T* alleles are expected to disrupt interactions with the catalytic center, suggesting that this part of Prp8, together with U6 and Cwc2, stabilize substrate positioning for the first-step catalysis (Fig. 4A). *prp8* alleles located further away from the catalytic center may act by a different mechanism. Although *prp8-F331L* was isolated from the original screen for *prp16-302* suppression, upon retesting, it was found to suppress *prp16-R686I* but not *prp16-302*. In contrast, *prp8-Y356C* suppresses *prp16-302* but not *prp16-R686I*. Whereas the location of *prp8-F331L* suggests its possible interactions with Snu114 (Brenner and Guthrie 2005), the mechanism of action of *prp8-D278G* and *Y356C* alleles needs further investigation.

In general, additivity of effects of combined alleles is observed when they individually affect the same process but act at different points or follow distinct mechanisms. Furthermore, combination of alleles that individually confer partial effects, acting by the same mechanism and affecting the same point in the process, will either lead to additivity (if they act in the same direction) or partial cancellation of effects (if they act in opposing directions). In contrast, the action of so-called “null” alleles, which by themselves are sufficient to fully exert a particular function, is not affected by combination with other null or partial alleles that act in the same process in the same direction. To test if the isolated *prp8* alleles located in the proximity of the catalytic center act through a single or different mechanisms, we prepared several double mutants and tested their effects on growth (Fig. 4D) and on suppression of *prp16-302* defects (Fig. 4F–H). Importantly, double mutants of *prp8* N-terminal domain *Y590F* + *K603I*, *K603I* + *K611I*, and *T589P* + *K611T* do not exert synthetic effects, as expected of alleles null for a function at a single event in the pathway (Fig. 4D). Each of the *prp8* alleles suppresses *prp16-302* defects (Fig. 4E) to a different extent, with *K603I* and *K611I/T* exerting the strongest suppression. Most of the tested combinations of mutant alleles, *Y590F* + *K603I*, *K603I* + *K611I*, and *T589P* + *K611T*, do not display additive effects consistent with their classification as null alleles (Fig. 4F–H). Only a combination of *T589P* + *K611I* alleles does not follow this pattern as they act additively both for suppression of *prp16-302* defects (Supplemental Fig. S1A) and for *ts* growth phenotypes (Supplemental Fig. S1B), consistent with their partial allele status.

Together, we identified multiple alleles of *cwc2*, U6, and *prp8* that suppress defects of *prp16-302* and thus facilitate the first-to-second steps transition. Some of the *cwc2* alleles are located in close proximity to the U6-_41_AACAAU_46_ motif, consistent with the described Cwc2–U6 interaction (Rasche et al. 2012). However, almost any disruption of the Cwc2 structure appears to correct *prp16-302 cs* defects, suggesting that overall stability/folding of Cwc2 is important for the function of the first-step catalytic center. Striking clustering of several *prp8* N-terminal alleles close to U6/*cwc2* mutants suggests their functional similarity.

### Effects of the U6, *cwc2*, and *prp8* alleles on splicing of suboptimal introns

**FIGURE 5.**
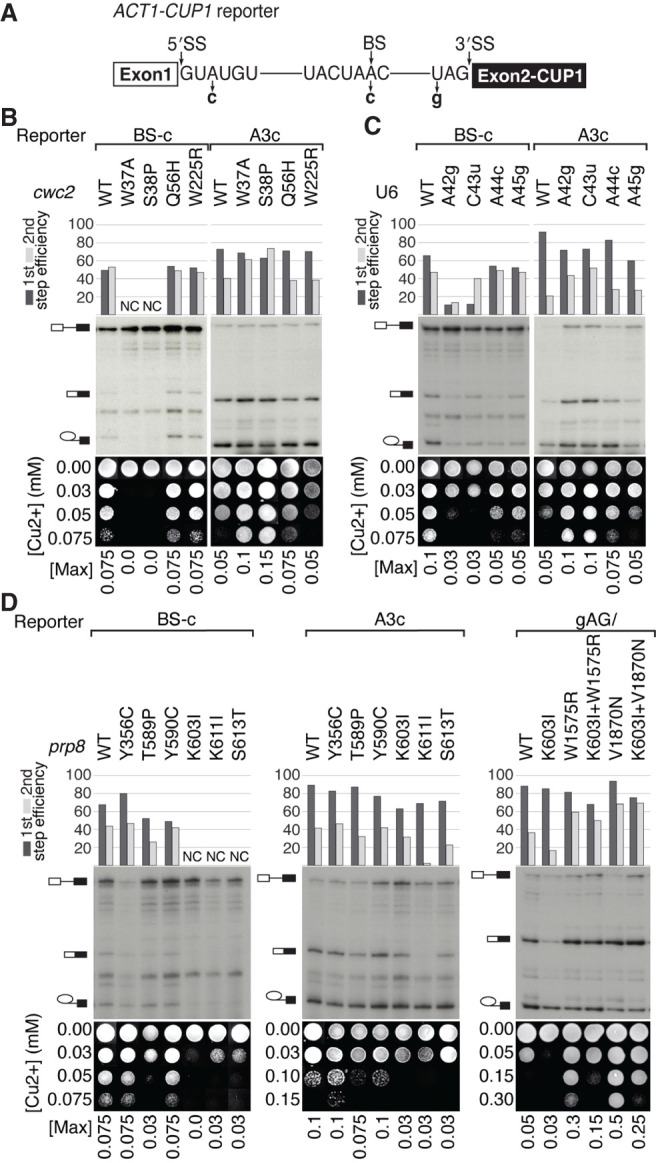
Effects of U6, *cwc2*, and *prp8* alleles on splicing of suboptimal introns. (*A*) Schematic of *ACT1–CUP1* reporter indicating the used 5′SS, BS, and 3′SS mutants. (*B*–*D*) Primer extension and copper growth assays for intron mutant reporters in strains carrying U6 (yMK20), *cwc2* (yMK79), or *prp8* (yJU75) alleles. (NC) Not calculated. (*B*) *cwc2*-*W37A* and *S38P* alleles exacerbate splicing defects of BS-c reporters (NC: bands corresponding to lariat-intermediate and spliced mRNA products are not detectable, preventing calculation of splicing efficiency) and improve the second step of splicing of A3c reporters. (*C*) U6-A42g and C43u alleles exacerbate the first step of splicing of the BS-c reporter while improving the second step of splicing of the A3c reporter (U6-A44c and A45g alleles display a similar, though very modest effect). (*D*) *prp8*-*T589P*, *K603I*, *K611I*, *S613T* alleles inhibit splicing of BS-c (for *K603I*, *K611I*, and *S613T*, alleles bands corresponding to lariat-intermediate and spliced mRNA products are not visible, preventing calculation of splicing efficiency) and A3c reporters. *prp8-V1870N* and *W1575R* second-step alleles improve splicing of gAG/reporters and *prp8*-*K603I* inhibits it. *prp8*-*K603I* allele opposes *prp8* second-step *W1575R* allele, diminishing its improvement of gAG/intron splicing. Only the first step of gAG/intron splicing is inhibited by *prp8*-*K603I* + *V1870N* allele compared to *prp8*-*V1870N* allele.

To further characterize the identified *prp16* suppressors, we analyzed their effects on splicing of suboptimal intron mutants defective in the first and/or second steps of splicing. Introns carrying an A–C mutation at the branch site (BS-c) are defective at both catalytic steps of splicing (Query and Konarska 2004), an A–C change at the intron position +3 (5′SS-A3c) inhibits transition to the second step (Konarska et al. 2006), and 3′SS-gAG/ mutation inhibits second-step catalysis (Fig. 5A; Collins and Guthrie 2001; Liu et al. 2007). Splicing of *ACT1–CUP1* reporters (Lesser and Guthrie 1993a) produces functional metallothionein Cup1, allowing Δ*CUP1* strains to tolerate the presence of copper proportionally to the efficiency of splicing.

To confirm the predicted role of Cwc2 stabilizing the first-step interactions, we analyzed splicing of intron reporters in yeast carrying various *cwc2* alleles (Fig. 5B). Among all *cwc2* alleles that suppress *prp16-302* defects, only *cwc2-W37A* and *S38P* significantly inhibit splicing of BS-c introns, as evidenced by reduced copper tolerance and reduced accumulation of the first-step products in primer extension analysis (Fig. 5B). However, the same alleles improve splicing of A3c introns, as shown by increased copper tolerance and increased accumulation of the second-step products in the corresponding primer extension results (Fig. 5B). Furthermore, within the spliceosome structure, these Cwc2 residues map near U6-AC42,43 (Fig. 2C), suggesting that destabilization of Cwc2–U6 interactions downstream from the 5′SS improves progression to the second step of splicing. Nonetheless, *cwc2* alleles do not significantly affect splicing of gAG/introns defective in the second catalytic step (Supplemental Fig. S2B).

All analyzed U6 suppressors of *prp16-302* defects inhibit splicing of BS-c introns, with U6-A42g and C43u exhibiting the strongest effects as evidenced by copper tolerance and primer extension (Fig. 5C). In contrast, the second step of splicing of A3c introns is strongly improved by U6-A42g and C43u, with a slight improvement seen for A44c and A45g (Fig. 5C). By analogy to *cwc2* effects, U6 upstream of ACAGA alleles do not significantly affect splicing of gAG/ reporters (Supplemental Fig. S2C). Thus, changes within U6-_41_AACAAU_46_ inhibit the first step of splicing, consistent with their *prp16-302* suppressor effect, with U6-A42g and C43u alleles particularly strongly destabilizing positioning of the 5′SS.

Finally, we analyzed *prp8* suppressor alleles for their effects on splicing. Four of them, *T589P*, *K603I*, *K611I*, and *S613T*, inhibit first and second steps of the splicing of BS-c reporters, as evidenced by reduced copper tolerance and reduced (*T589P*) or not detectable (*K603I*, *K611I*, and *S613T*) accumulation of splicing products in primer extension analysis (Fig. 5D). Additionally, *prp8-Y590C* slightly inhibits the first step of splicing of BS-c reporters, as seen in primer extension results, but this inhibition is not detectable by copper assay (Fig. 5D). The strongest inhibition was observed for *K603I*, *K611I*, and *S613T* alleles, located in proximity to the active site (Fig. 4A). In contrast to U6 and *cwc2* alleles that improve the second step for A3c reporters, the tested *prp8-T589P*, *K603I*, *K611I*, and *S613T* alleles inhibit it, as evidenced by copper assays and primer extension analyses, with *K611I* exhibiting the strongest effect (Fig. 5D). We also analyzed the effect of *prp8* N-terminal *K603I* allele on splicing of the 3′SS-gAG/reporter defective for the second step. Whereas *prp8* second-step alleles *V1870N* and *W1575R* improve splicing of the 3′SS-gAG/introns (Liu et al. 2007), N-terminal *K603I* allele inhibits the second step and diminishes the second-step improvement of *prp8-W1575R* allele (*K603I* + *W1575R*) (Fig. 5D). The analogous combination of *prp8-K603I* with the strongest second-step allele, *prp8-V1870N*, only slightly inhibits the first step (*K603I* + *V1870N*).

Whereas the analyzed U6 and *cwc2* alleles inhibit only the first step of splicing by destabilizing the positioning of the 5′SS in the first catalytic conformation, *prp8* alleles appear to inhibit both the first and second steps by destabilizing the interactions within the catalytic core of the spliceosome.

### How is the first-to-second-step transition affected by various alleles in U6 and Prp8?

To understand the effects of the isolated suppressor alleles on the catalytic center during the transition between the catalytic steps, we combined several *prp8* N-terminal alleles with U6 alleles, shown to suppress or exacerbate *prp16-302* defects (Eysmont et al. 2019). Combining different suppressor alleles can be used to determine their individual contributions to an analyzed process. Thus, to establish if the characterized *prp8* alleles follow the same or distinct mechanisms from different classes of U6 alleles, we analyzed these alleles in pairwise combinations.

**FIGURE 6.**
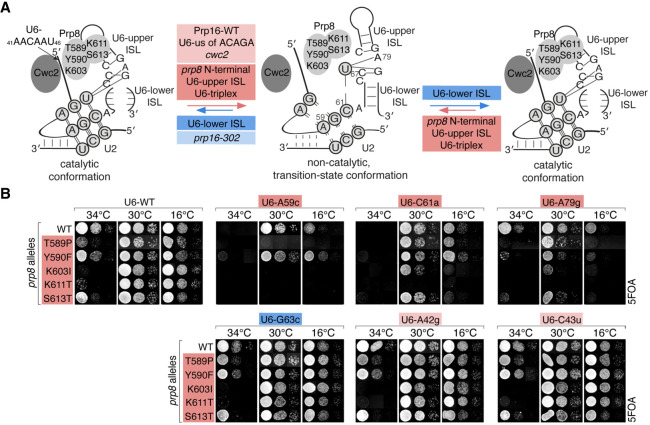
Interactions between various classes of U6 and *prp8* N-terminal alleles. (*A*) The model of rearrangements of the catalytic center between the two catalytic steps. Four classes of alleles are marked by colored boxes: those promoting exit from the first-step catalytic conformation and entry into the transition-state conformation (light red); those promoting entry into the transition-state conformation through destabilization of both catalytic steps (red); those inhibiting exit from the first-step catalytic conformation (light blue), and those promoting both catalytic conformations (blue). (*B*) Genetic interactions between *prp8*-*T589P*, *Y590F*, *K603I*, *K611T*, *S613T* and U6-A59c, C61a, A79g, G63c, A42g, C43u alleles in *prp8*Δ U6Δ strain (yCQ05) tested by growth in the presence of 5FOA. *prp8*-*T589P*, *K603I*, *K611T*, *S613T* alleles become synthetically lethal in combination with U6-A59c. U6-C61a and A79g become synthetically lethal with *prp8-K611T* allele or *cs* and/or *ts* in combination with *prp8*-*T589P*, *Y590F*, *K603I*, *S613T* alleles. *prp8*-*T589P*, *Y590F*, *K603I*, *K611T* alleles do not affect growth in combination with U6-G63c, A42g, C43u, compared to wt U6.

Individually, U6-A59c, C61a, and A79g alleles destabilize the catalytic triplex directly (A59 and C61 form the catalytic triplex) or indirectly (A79 is adjacent to the triplex-forming U80), and thus suppress *prp16-302* defects by destabilizing the catalytic conformation (shown in red, Fig. 6A; Table 1; Eysmont et al. 2019). U6-A59c allele becomes synthetically lethal in combination with most of the *prp8* N-terminal alleles *T589P*, *K603I*, *K611T*, and *S613T* (Fig. 6B). Similarly, U6-C61a or U6-A79g alleles become synthetically lethal with *prp8-K611T* allele or *ts* and/or *cs* in the presence of *prp8-T589P*, *Y590F*, *K603I*, and *S613T* alleles (Fig. 6B).

**TABLE 1.** Alleles affecting transitions between the two catalytic conformations of the spliceosome

	Name	Allele	Source
First-step catalytic conformation	*prp16-302*	*R456K* and *G691R*	Madhani and Guthrie 1994
	*prp16-R686l*	*R686l*	Hotz and Schwer 1998
	U6-lower ISL	G63c	Eysmont et al. 2019
Transition-state suppressors of *prp16-302*	U6-upper ISL	A79g	Eysmont et al. 2019
	U6-catalytic triplex	A59c, C61a	Eysmont et al. 2019
	U6-upstream of ACAGA	ΔA41, A41c, A41g, A42c, A42g, ΔC43, C43g, C43u, ΔA44, A44c, A44u, A45g, U46a, U46g	Madhani and Guthrie 1994; this study
	*cwc2*	*W37A*	Hogg et al. 2014
		*E17D, S38P, Q54L, P55S, Q56H, D98V, K101T, C111R, l127T, N134S, F162l, W225R*	This study
	*prp8* N-terminal	*D278G, F331L, Y356C, T589P, Y590F, Y590C, K603l, K611L, K611l, K611T, K611E, S613T*	This study
Second-step catalytic conformation	U6-lower ISL	G63c	Eysmont et al. 2019
	*prp8* second step	*V1870N, W1575R*	Liu et al. 2007

These results suggest that the analyzed U6-triplex mutants and *prp8* alleles affect the catalytic center through distinct mechanisms, which, when combined, result in synthetic, synergistic effects. In contrast, the combination of *prp8* N-terminal alleles that disrupt catalytic conformations and the U6-G63c lower ISL allele (Fig. 6B) that promotes catalytic conformations leads to cancellation of effects, yielding no significant growth phenotypes (red and blue, Fig. 6A; Table 1).

In contrast to U6-triplex mutants, U6-A42g and C43u alleles that destabilize the first-step conformation and suppress *prp16-302* defects (Madhani and Guthrie 1994), do not affect cell growth when combined with *prp8* N-terminal domain alleles (Fig. 6B), suggesting that these U6 alleles disrupt catalytic conformation by the same, or similar mechanism to that used by *prp8* N-terminal alleles (light red and red, Fig. 6A). We note one observed exception to this rule: *prp8-T589P* in combination with U6-A42g + C43u exerts a stronger *ts* phenotype than either of the alleles by themselves (Supplemental Fig. S1C).

We conclude that the analyzed *prp8* alleles favor the transition-state conformation by destabilizing both flanking catalytic conformations. These *prp8* alleles may affect the first-step conformation acting similarly to U6 and *cwc2* alleles, by destabilizing the 5′SS positioning. The same alleles also inhibit the second step by destabilizing different interactions at the catalytic center.

## DISCUSSION

Although the catalytic center of the spliceosome has been extensively studied in a variety of systems and by diverse techniques, the mechanisms supporting its function are largely unknown. RNA-dependent ATPases/helicases facilitate important transitions in the spliceosome assembly, catalysis, release, and recycling. In particular, Prp16 modulates the transition between the two catalytic reactions of splicing, and its mutant alleles, e.g., *prp16-302,* serve as useful markers of this transition. Analysis of suppressor alleles that correct *prp16-302* growth defects can therefore explain in part mechanisms involved in the exit from the first-step catalytic conformation and transition into the second step of splicing.

To better understand the nature of such changes at the transition between the two catalytic steps, we characterized three groups of suppressor alleles that correct *prp16-302* growth defects: strong U6 suppressor alleles identified by Madhani and Guthrie (1994) (Fig. 3C), and two groups of suppressor alleles identified in genetic screens: in *cwc2* (Fig. 2D) and *prp8* (Fig. 4B). Mutant alleles of all these factors disrupt catalytic interactions and, by supporting Prp16 action, facilitate exit from the first-step catalytic conformation.

### Characterization of U6, *cwc2*, and *prp8* suppressor alleles

Previously, we identified *prp16* suppressor alleles in the lower part of U6-ISL—these mutants destabilize the U6-ISL structure, allowing exit from the first-step catalytic conformation (Eysmont et al. 2019). Other suppressors of *prp16* defects, e.g., *cwc2* (Hogg et al. 2014), *ecm2* (van der Feltz et al. 2021), U6 (Madhani and Guthrie 1994; Fica et al. 2014), *isy1* (Villa and Guthrie 2005), and U2 alleles (Hilliker et al. 2007; Perriman and Ares 2007; Mefford and Staley 2009), have been identified; however, how they improve exit from the first-step conformation is not known.

Here, we focused on the U6-_41_AACAAU_46_ region bordering the U6-_47_ACAGA_51_ motif, where Madhani and Guthrie (1994) have found multiple alleles correcting *prp16-302 cs* growth defects. We confirmed and extended these findings by identifying a large cluster of U6 alleles that suppress *prp16-302* defects, improving exit from the first-step catalytic conformation (Fig. 3). Next, based on biochemical data (Rasche et al. 2012; Schmitzova et al. 2012) and cryo-EM structures (5LJ5) (Galej et al. 2016), we analyzed Cwc2, a component of the NTC complex that interacts with U6-_41_AACAAU_46_ as well as with intron nucleotides downstream from the 5′SS. We identified multiple *cwc2* alleles that, like U6-_41_AACAAU_46_ alleles, correct growth defects of *prp16* mutants (Fig. 2). The strongest effects were observed for *cwc2-W37A* and *S38P* alleles located in proximity of U6-_41_AAC_43_, that likely disrupt Cwc2–U6 interactions and destabilize positioning of the 5′SS for the first step. The remaining *cwc2* suppressors do not contact U6 directly and most likely act by generally destabilizing the protein, altering its interactions with other spliceosomal factors known to suppress *prp16* defects, e.g., Ecm2 and Isy1 (Villa and Guthrie 2005; Hogg et al. 2014; van der Feltz et al. 2021).

Suppression of *prp16* defects by *cwc2* alleles places their action at the exit from the first-step conformation, facilitated by Prp16 ATPase. This notion is further supported by effects of these *cwc2* alleles on *prp2-Q548N*, defective in promoting entry into the first-step conformation (Fig. 2A,G). Although wt Cwc2 is not required in vitro for Prp2-dependent remodeling of the activated spliceosome (Rasche et al. 2012), our results show that destabilization of the first-step interactions by *cwc2* mutants inhibits Prp2 action (Fig. 2G). Both *cwc2*-*W37A* and *Q54L* alleles strongly exacerbate defects of the *prp2*-*Q548N* allele even in the presence of wt endogenous Cwc2. We conclude that Cwc2 promotes spliceosome activation mediated by Prp2 ATPase, which facilitates transition into the first-step conformation and activates the spliceosome for the first-step catalysis (Kim and Lin 1993, 1996; Bao et al. 2017). This confirms that *cwc2* alleles affect the spliceosome conformation flanked by actions of Prp2 and Prp16 and thus destabilize the first-step spliceosomal conformation.

Independently, we analyzed Prp8, a protein forming extensive contacts with the entire catalytic core of the spliceosome, for alleles that suppress *prp16-302* defects. The N-terminal domain of Prp8 directly contacts Cwc2 and U6 snRNA (5LJ5) (Galej et al. 2016), and several *prp8* alleles located in proximity of U6 and *cwc2* alleles (*prp8*-*T589P*, *Y590C/F*, and *K603I*), or the active site (*K611L/T/I/E* and *S613T*) (Fig. 4A), are likely to affect not only the 5′SS positioning but also U6-ISL and other catalytic interactions. The remaining *prp8* alleles (*D278G*, *F331L*, and *Y356C*) may destabilize catalytic conformations by altering Prp8 interactions with other spliceosomal components, e.g., Snu114 (Brenner and Guthrie 2005).

*prp8* alleles exhibiting the strongest splicing effects are clustered in proximity of the U6:Cwc2 interaction and U6-ISL (*prp8-T589*, *Y590*, *K603*, *K611*, and *S613*) (Fig. 5D). Interestingly, all of these amino acid positions (except for T589) are highly conserved in evolution (Supplemental Fig. S3). One of the *Caenorhabditis elegans* alleles altering the cryptic splice site selection, *az43*, corresponds to *S. cerevisiae prp8-T607S*, which is inviable in yeast (Mayerle et al. 2019). Prp8 residues T607, K611, and S613 lie within a short α-helix adjacent to U6-_42_ACA_44_ upstream of the 5′SS-binding site, and U6-ISL adjacent to the catalytic triplex, as well as intron position G1, suggesting close interactions of the corresponding alleles with important elements of the catalytic center.

### Mechanisms of *prp16-302* suppression by U6, *cwc2*, and *prp8* alleles

Juxtaposition of U6 and *cwc2* alleles immediately downstream from the 5′SS suggests that U6–Cwc2 interactions stabilize the first-step conformation by positioning 5′SS for catalysis. However, despite close proximity of some U6, *cwc2*, and *prp8* alleles they exhibit some important differences, raising questions whether they act by the same or distinct mechanisms and/or affect the same or different events in the process. Analysis of several N-terminal *prp8* alleles suggests that they display features of null alleles: Most of the tested combinations of these alleles exhibit a characteristic lack of additivity in suppression of *prp16-302* defects (Fig. 4F–H) or exacerbation of *ts* growth phenotype (Fig. 4D). The notable exception is the combination of *prp8-T589P* and *K611I* mutants, which exhibits a *ts* phenotype (Supplemental Fig. S1B) and suppresses *prp16-302* defects more strongly than either allele alone (Supplemental Fig. S1A), suggesting that they either represent partial function alleles or act through different mechanisms. Interestingly, the *prp8-T589P* + *K611T* combination does not additively suppress *prp16-302* defects as compared to its individual alleles (Fig. 4H). This amino acid specificity of effects suggests that position K611 is involved in more than one functional interaction.

Based on our analyses, we conclude that at least *prp8-K603I* and *K611T* fulfill the criteria of null alleles. This allowed us to analyze combinations of *prp8* and U6 alleles that individually suppress *prp16-302* defects to establish if they act by the same or distinct mechanisms. In combination with U6 mutants of the catalytic triplex, all of the tested *prp8* alleles showed additive effects, consistent with the notion that U6 catalytic triplex mutants destabilize catalytic conformation differently from N-terminal *prp8* mutants. In contrast, combinations of *prp8* alleles with U6 mutants upstream of ACAGA motif showed no detectable additivity. *prp8-T589P* is the only *prp8* allele that exhibits modest additivity of *ts* phenotype in combination with U6-A42g + C43u double mutant (Supplemental Fig. S1C), consistent with its status of a partial function allele. Thus, U6 mutants upstream of ACAGA and *prp8* alleles likely share the same mechanism to destabilize the first-step interactions. However, because these U6 alleles do not display any growth phenotypes on their own, this conclusion cannot be firmly established.

Hyperstable pairing of the 5′SS-A3c to U6-G50 (Konarska et al. 2006) interferes with the first-to-second-step transition, suggesting that transient destabilization of the 5′SS-U6 contacts is needed to exit the first step. Indeed, destabilization of U6–Cwc2–intron interactions facilitates splicing of 5′SS-A3c introns (Fig. 5B,C), specifically affecting the first, but not the second-step conformation (Supplemental Fig. S2). In contrast, N-terminal *prp8* alleles inhibit both steps of splicing, perhaps by affecting not only positioning of the 5′SS but also contacting U6-ISL and thus interfering with overall rearrangements at the catalytic center. These results support the available cryo-EM structural information (Galej et al. 2016; Fica et al. 2017), suggesting that U6–Cwc2–5′SS interactions during the two catalytic conformations are unchanged, but argue that they do not significantly affect the stability of the second-step conformation.

The previously identified second-step *prp8* alleles (Collins and Guthrie 1999; Query and Konarska 2004; Liu et al. 2007) also facilitate the transition from the first-to-second steps and could thus be considered to act similarly to the N-terminal domain alleles. However, none of these second-step alleles suppress *prp16-302* defects. Furthermore, whereas second-step alleles improve the second step, the N-terminal alleles inhibit both catalytic steps, indicating that they act by different mechanisms.

Alleles in three spliceosomal factors: U6, Cwc2, and Prp8, that suppress defects of the *prp16-302* allele, identified important catalytic interactions stabilizing the first-step conformation but destabilized by the Prp16 ATPase at the transition to the second step. Our results suggest that in the first step, the cluster of closely positioned U6, *cwc2*, and *prp8* alleles appears to act jointly to destabilize binding of the 5′SS at the catalytic center. However, despite the similarity of the overall action, *cwc2* and U6 alleles destabilize just the first-step catalytic conformation, whereas the identified *prp8* alleles destabilize both the first and second catalytic steps. Thus, if Cwc2 and U6 interactions are present in the second-step conformation (as indicated by cryo-EM structures) (Fica et al. 2017), these contacts are unlikely to contribute to the stability of the second-step catalytic center.

In addition to altering the 5′SS positioning, *prp8* alleles seem to also influence contacts with U6-ISL, interfering with overall rearrangements at the catalytic center at both the first and second steps of splicing. The identified *prp8* alleles act to destabilize both catalytic conformations of the spliceosome, indirectly informing us about the features of the transition-state conformation that needs to form between the two catalytic steps (Fig. 6A). The existence of this conformation is deduced from genetic experiments and supported by biochemical studies (Bao et al. 2017), but to date are not confirmed by cryo-EM structural analyses. Our genetic analysis of *prp16* suppressor alleles may help to understand the transition-state conformation, possibly leading to its future structural analysis.

## MATERIALS AND METHODS

*S. cerevisiae* strains used in this study are described in Supplemental Table S1.

### Plasmid-shuffle growth assays

Cell cultures were adjusted to OD_600_ = 1 and 10-fold serial dilutions were spotted on plates containing 5FOA, grown at indicated temperatures, and photographed after 3–6 days.

### Copper growth assays

yMK20, yMK79, or JU75 strains carrying indicated alleles were transformed with *LEU*-marked *ACT1–CUP1* reporter plasmids (Lesser and Guthrie 1993a). Cultures were grown in –LEU medium diluted to *A*_600_ = 1.0, and equal volumes were spotted on –LEU plates containing 0–1.0 mM CuSO_4_ and grown at 30°C for 4 days. Data for each experiment, cropped and aligned for clarity, originate from a single plate.

### Primer extension assays

Total RNA was isolated from strains carrying *ACT1–CUP1* reporter plasmids using glass beads and a standard phenol:chloroform protocol. Primer complementary to the second exon of *ACT1* (5′-GGCACTCATGACCTTC-3′) was kinased at the 5′ end using T4 PNK and γ-^32^P ATP (Hartmann, SRP-501). Annealing (containing 3 μg of total yeast RNA and ^32^P-labeled primer) and extension reactions were carried out as described by Eysmont et al. (2019).

### Quantitation of splicing efficiency

To calculate first and second steps splicing efficiency, images of radiography films were analyzed using the ImageJ tool, resulting in the intensity of each primer extension product. First-step efficiency was calculated as the intensity of the first-step products (lariat intermediates + mRNA)/total RNA (lariat intermediates + mRNA + pre-mRNA). Second-step efficiency was calculated as the intensity of the second-step product (mRNA)/first-step products (lariat intermediates + mRNA).

### Bioscreen C growth measurements

Cell cultures were adjusted to OD_600_ = 0.1 and transferred in 300 μL triplicates to a 100-well Honeycomb 2 Microplate. Cultures were grown at 16°C or 18°C with continuous, double-orbital shaking in the Bioscreen C Pro system. OD_600_ measurements were taken at regular intervals of 15 min over 140 h. Growth curves were generated using the average of three repeats.

### Screen for *cwc2* suppressors of *prp16-302*

To increase the analyzed pool of mutants, we carried out the selection in parallel for two regions of Cwc2 (corresponding to aa 1–159, flanked by SnaBI and EcoRI sites, and aa 159–339, flanked by EcoRI and PflMI). In vivo gap repair selection for improvement of *prp16-302 cs* growth defects was carried out at 16°C. Error-prone PCR was performed using a buffer containing 0.5 mM MnCl_2_, 5.5 mM MgCl_2_, and 0.05 U/µL of *Taq* DNA polymerase.

### Screen for *prp8* N-terminal domain suppressors of *prp16-302*

To increase the number of tested mutants, mutagenesis of the N-terminal Prp8 domain was carried out in four regions using NheI, BamHI, SalI, SexAI, and SacI restriction sites, covering nucleotides +1 to +477 or +477 to +1017 of *PRP8*. In vivo gap repair selection for improvement of *prp16-302 cs* growth defects was carried out as for *cwc2* suppressors.

## SUPPLEMENTAL MATERIAL

Supplemental material is available for this article.

## Figures and Tables

**Jadwiga Meissner:**
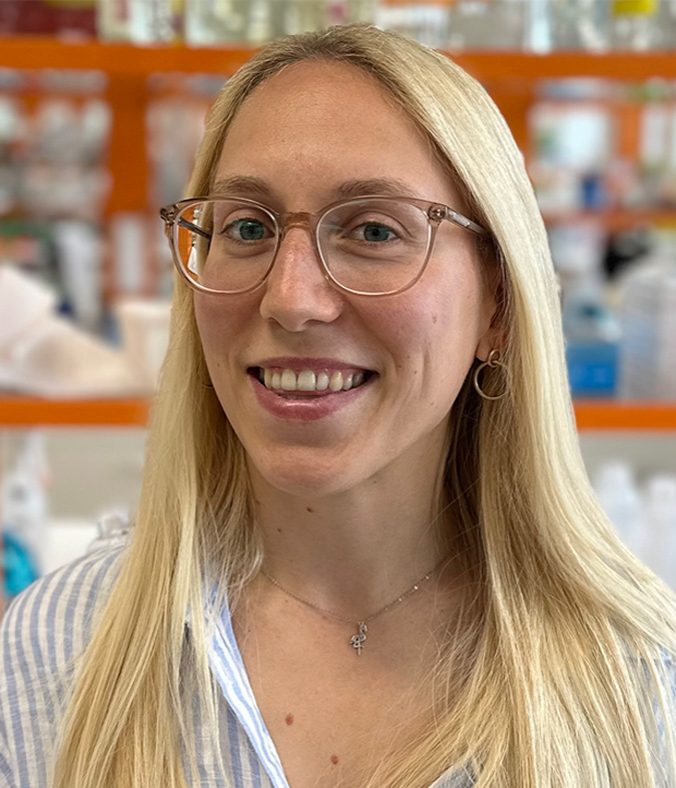


**Katarzyna Eysmont:**
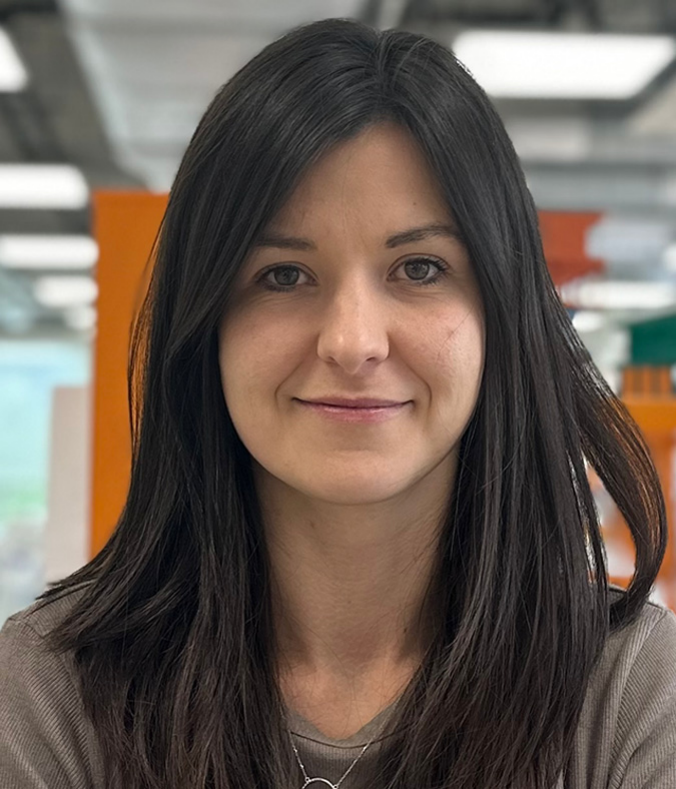


## MEET THE FIRST AUTHORS

***Meet the First Author(s)* is an editorial feature within *RNA*, in which the first author(s) of research-based papers in each issue have the opportunity to introduce themselves and their work to readers of *RNA* and the RNA research community. Jadwiga Meissner and Katarzyna Eysmont are co-first authors of this paper, “Characterization of Cwc2, U6 snRNA, and Prp8 interactions destabilized by Prp16 ATPase at the transition between the first and second steps of splicing.” Jadzia is a graduate student and Kasia is a postdoctoral researcher at the Konarska Laboratory of RNA Biology at IMol, a new international institute of the Polish Academy of Sciences. Their major research focuses on understanding the mechanism of splicing.**

**What are the major results described in your paper and how do they impact this branch of the field?**

Our studies focus on understanding the mechanisms by which the spliceosome, a molecular machine responsible for pre-mRNA splicing, transitions between its catalytic steps.

We identify multiple alleles in Cwc2, U6 snRNA, and Prp8 that suppress the defects of the *prp16-302* allele in *Saccharomyces cerevisiae*. These alleles were found to destabilize the first-step catalytic conformation, facilitating the transition to the second step of splicing. These findings significantly contribute to the field of RNA splicing by elucidating the mechanisms of spliceosome rearrangements and identifying key factors that modulate transition-state conformation of the spliceosome.

**What led you to study RNA or this aspect of RNA science?**

**KE:** My journey into studying RNA began during my undergraduate studies at Warsaw University, where I was introduced to the RNA World by studying the RNA exosome in Professor Joanna Kufel's laboratory. There, I started to understand the importance and versatility of RNA in biological systems, its multifunctional nature, and its immense potential for scientific and medical advancements.

**JM:** My interest in RNA science began during my college years, when I studied molecular biology and became fascinated by how cellular processes are dictated by the genome. This naturally led to my interest in posttranscriptional modifications of RNA and the intricate process of splicing. At that time, I was fortunate to learn about these topics from my mentor, Magda Konarska, a pioneering figure in the splicing field with numerous contributions to the RNA community. What drives my interest in splicing mechanisms is the opportunity to dive into the molecular world and discover the workings of such a complex machine as the spliceosome.

**During the course of these experiments, were there any surprising results or particular difficulties that altered your thinking and subsequent focus?**

The input from reviewers of our paper provided invaluable insights that pushed our analysis of the study's alleles to a significantly higher level. Their feedback motivated us to explore the underlying mechanisms of these alleles more thoroughly, enhancing our knowledge of their impact on the catalytic center of the spliceosome. A particularly surprising finding was the difference between U6 and *cwc2* alleles as compared to *prp8* alleles. While all these factors interact to stabilize the first-step catalytic conformation, only *prp8* alleles impact the second-step conformation. This discovery significantly clarified our concepts concerning rearrangements within the catalytic core of the spliceosome.

**What are some of the landmark moments that provoked your interest in science or your development as a scientist?**

**KE:** My initial interest in science was sparked by a high school biology class in Warsaw, where I first learned about the central dogma of molecular biology (DNA → RNA → Protein). The idea that genetic information flows in such a precise and regulated manner fascinated me and ignited my curiosity about molecular processes. A few years later, my internships at Aarhus University in Denmark and at Rockefeller University in New York City were among the most formative experiences in my scientific journey. Being surrounded by dedicated and passionate researchers was incredibly inspiring and deepened my commitment to pursuing a career in scientific research.

**JM:** One of the landmark moments that sparked my interest in science, particularly in the field of splicing, was the publication of the first cryo-EM spliceosome structures. It was incredible to see the detailed structure that aligned so well with years of biochemical and genetic analyses. Witnessing how accurately the splicing community had predicted the intricate workings of this complex machinery without previously seeing its structure was inspiring. This milestone motivated me to learn from these structures and to explore how we can go beyond the structural data to uncover even more about the inner workings of the spliceosome.

**If you were able to give one piece of advice to your younger self, what would that be?**

**KE:** Looking back, I would urge my younger self not to be afraid to ask questions, no matter how simple or complex they may seem. I would further say, stay persistent, keep an open mind, and always seek out mentors and collaborators who can guide and inspire you.

**JM:** Decisions are not permanent. Don't be afraid to make a choice, start something new, or take a path you're unsure about. You can always change direction if the initial choice doesn't work out. Embrace the journey and be open to course changes along the way.

**Are there specific individuals or groups who have influenced your philosophy or approach to science?**

**KE:** Two women, Professors Joanna Kufel and Magda Konarska, profoundly shaped my approach to science. They emphasized the importance of curiosity, persistence, and continual learning. I am deeply grateful for their influence in shaping me both as a scientist and as an individual.

**JM:** My scientific philosophy and approach have been profoundly shaped by Magda Konarska, whose lab I joined early in my bachelor's studies. She introduced me to the world of science, sharing her lifelong fascination with splicing. Her passion for uncovering the mechanistic details of the spliceosome continues to inspire me daily and has shaped my own dedication to seeking the mechanisms behind biological processes.

**What are your subsequent near- or long-term career plans?**

**KE:** My near-term career plans include continuing my research in RNA splicing, focusing on publishing my findings and expanding my expertise. In the long term, I aim to find my own niche within the RNA biology field and establish a research program that will significantly impact our understanding of RNA mechanisms and their applications.

**JM:** As I approach the defense of my PhD, I am considering the important decisions that lie ahead for my career. I am currently weighing whether to stay in academia to continue unraveling the mechanistic intricacies of life, or to transition into the industry, where I can apply my expertise to more practical, translational projects.

**What were the strongest aspects of your collaboration as co-first authors?**

When Jadzia first joined the lab, Kasia guided her through the intricacies of lab work and experimental techniques. This mentorship laid the foundation for a strong relationship both in and out of the lab. Our collaboration was seamless due to our complementary strengths: Kasia's focus, organization, and problem-solving skills, combined with Jadzia's exploratory approach to scientific questions. Throughout the project, we provided each other with continuous support and encouragement. This emotional and intellectual backing was crucial for maintaining our morale and enthusiasm, especially during challenging phases of the project. We tackled technically difficult tasks, like primer-extension analysis, by alternating turns, which made managing these challenges more effective.

**How did you decide to work together as co-first authors?**

The project was originally started by Kasia, who conducted most of the experiments. She then took a maternity leave break, during which Jadzia continued the project with great dedication. After navigating many pivotal moments and setbacks, Jadzia successfully completed the project and drafted the manuscript. At a crucial moment, following the first review, we decided to work together to address all the suggestions. This was an intense period, marked by technical problems with the experiments, but we combined our individual strengths and insights, ultimately enhancing the quality of the research and accelerating the process. It was natural for us to be co-first authors, without any doubt, and we are certain that without this collaboration, it would not have been possible.
